# Down syndrome birth weight in England and Wales: Implications for clinical practice

**DOI:** 10.1002/ajmg.a.37366

**Published:** 2015-09-26

**Authors:** Joan K. Morris, Tim J. Cole, Anna L. Springett, Jennifer Dennis

**Affiliations:** ^1^ Wolfson Institute of Preventive MedicineQueen Mary University of LondonLondonUnited Kingdom; ^2^ Population, Policy and Practice ProgrammeUCL Institute of Child HealthLondonUnited Kingdom; ^3^ Down Syndrome Medical Interest GroupOxfordUnited Kingdom

**Keywords:** Down syndrome, trisomy 21, birth weight, gestational age, growth charts

## Abstract

The aim of this study was to determine if syndrome‐specific birth weight charts were beneficial for babies with Down syndrome in England and Wales. Birth weights of 8,825 babies with Down syndrome born in England and Wales in 1989–2010 were obtained from the National Down Syndrome Cytogenetic Register. Birth weight centiles for 30–42 weeks gestation by sex were fitted using the LMS method and were compared to those for unaffected babies from the UK‐WHO growth charts. For babies born with Down syndrome the median birth weight from 37 to 42 weeks was 2,970 g (10th–90th centile: 2,115–3,680) for boys and 2930 g (2,100–3,629) for girls, and the modal age of gestation was 38 weeks, 2 weeks earlier than for unaffected babies. At 38 weeks gestation they were only slightly lighter than unaffected babies (159 g for boys and 86 g for girls). However at 40 weeks gestation the shortfall was much greater (304 g and 239 g, respectively). In neonates with Down syndrome there is little evidence of growth restriction before 38 weeks gestation, so up to this age it is appropriate to use the UK‐WHO birth weight charts. Thereafter birth weight is below that of unaffected babies and it should be plotted on the UK Down syndrome growth charts. © 2015 The Authors. *American Journal of Medical Genetics Part A* Published by Wiley Periodicals, Inc.

## INTRODUCTION

Growth charts show that early in life babies with Down syndrome gain weight more slowly than unaffected babies [Piro et al., 1990; McCoy, 1992; Myrelid et al., 2002; Styles et al., 2002]. However there is a lack of data on their prenatal growth. Since the seminal paper of Smith and McKeown [1955], using cross sectional birth weight data as a proxy measure of intrauterine growth in late pregnancy, only two others [Clementi et al., 1990; Boghossian et al., 2012] have presented data on the birth weight of babies with Down syndrome according to gestational age at birth. It is nevertheless a belief widely held that among those born with Down syndrome there is an excess of preterm birth and low birth weight [Cunningham, 2006]. Our study used cross sectional birth weight data from the National Down Syndrome Cytogenetic Register (NDSCR) to test this belief and to determine whether syndrome‐specific birth weight charts are necessary for this population.

## METHODS

The NDSCR was set up on January 1st 1989, and currently holds anonymous data on over 33,000 ante‐ or postnatal diagnoses of Down syndrome obtained from all clinical cytogenetic laboratories in England and Wales [Mutton et al., 1991]. It has approval from the Confidentiality Advisory Group, under Section 251 of the NHS Act 2006, to collect, process and use data without the need for individual consent. It also has ethics approval from the Trent Medical Research Ethics Committee (MREC).

Virtually every baby with clinical features suggesting Down syndrome, and any antenatal diagnostic sample from a pregnancy suspected to have Down syndrome, receives a cytogenetic examination because the definitive test for the condition is the finding of trisomy 21. The data in the register are obtained from all clinical cytogenetic laboratories in England and Wales, which are requested to send a completed form for each such diagnosis and its variants. The form contains details of the date, place of birth, and indications for referral, maternal age, and family history. Most laboratories send a copy of this form to the referring physician for confirmation and completion. The gestational age was estimated from the date of the last menstrual period (LMP), and was usually confirmed by ultrasound.

Comparisons with other congenital anomaly registers and the Office for National Statistics show that since its inception the register has captured data on an estimated 93% of all diagnosed Down syndrome births and pregnancy terminations for residents of England and Wales [Savva and Morris, 2009].

Centiles were fitted to the birth weight data with the LMS method [Cole and Green, 1992] in R (http://www.R-project.org/) using the gamlss package [Rigby and Stasinopoulos, 2005]. The Box–Cox–Cole–Green (BCCG) distribution was used with a log link for the median. In essence the LMS method estimates for each week of gestation the median birth weight (M) and its coefficient of variation (S), allowing for non‐normality in the distribution by using a Box–Cox power transformation (L). The sex difference in median birth weight did not vary with gestation and so was fitted as a constant, this (due to the log link) corresponding to a constant percentage difference. The centiles were fitted using data from 28 to 43 weeks of gestation, and are presented from 30 to 42 weeks as tables of smoothed L, M, and S values by sex. From these values, centiles C_100__α_ were derived using the formula

$$C_{100\alpha }={M(1+L\times S\times z_{\alpha })}^{1/L}$$

where z_α_ is the standard deviation for tail area α under a Normal distribution. This leads to approximate 2nd, 9th, 25th, 50th, 75th, 91st, and 98th centiles using the two‐thirds of a standard deviation spacing proposed by Cole [1994].

The birth weight centiles were compared with those for unaffected babies from the revised UK‐WHO growth charts [Cole et al., [Link]], which were based on 9,443 babies with gestational age estimated by date of LMP confirmed by ultrasound.

The secular trend in birth weight was examined using linear regression of birth weight SD score on year of birth. The association between gestational age and missing birth weight was estimated using logistic regression.

## RESULTS

**Figure 1:**
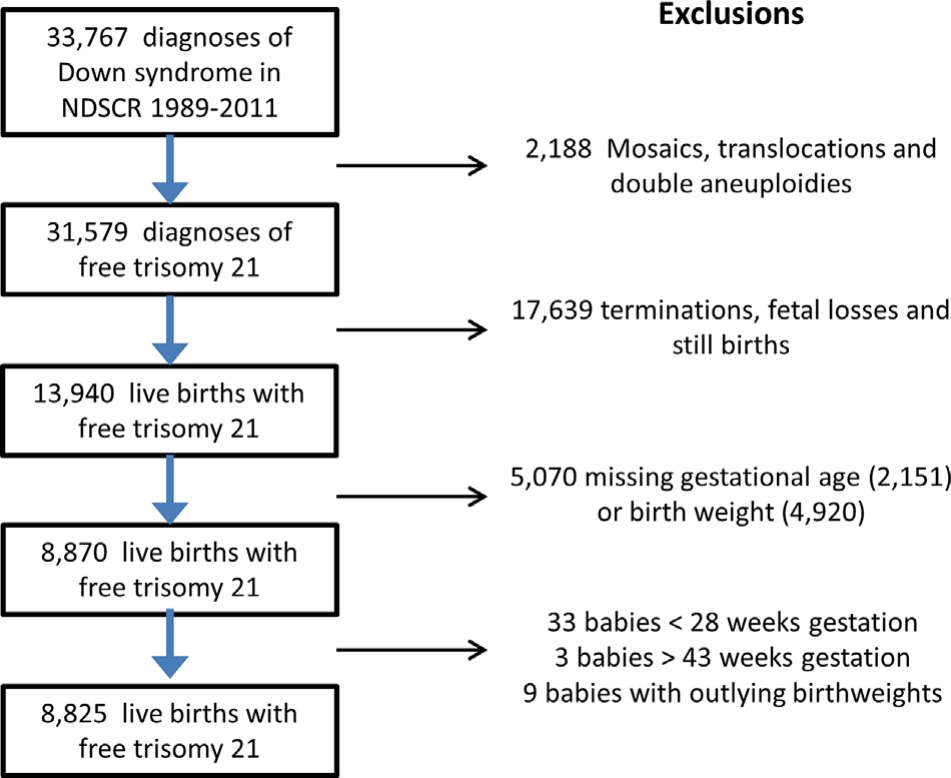
Flow chart of the selection of free trisomy 21 cases for inclusion in the analysis. [Color figure can be seen in the online version of this article, available at http://wileyonlinelibrary.com/journal/ajmga].

33,767 diagnoses of Down syndrome were recorded in the register from January 1st 1989 to December 31st 2011, from which 8,825 live births with free trisomy 21 and complete information on birth weight and gestational age were extracted (see flow chart in Fig. 1).

**Figure 2:**
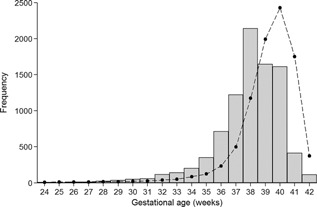
The distribution of gestational age at birth in babies with Down syndrome (gray bars) compared to unaffected babies (dashed line) [Office for National Statistics, 2012].

Figure 2 shows the distribution of gestational age at birth for babies with Down syndrome compared to unaffected babies born in England and Wales in 2010 [Office for National Statistics, 2012], scaled to adjust for the different sample sizes. The modal gestational ages were 38 weeks for Down syndrome babies and 40 weeks for unaffected babies.

Table I gives the sample sizes and fitted LMS parameters for birth weight by sex and gestational age. The skewness (L) and coefficient of variation (S) parameters were the same by sex, while median birth weight (M) was 2.4% (95%CI: 1.7–3.1) less in girls than boys at all gestations. There was no evidence of a secular trend in birth weight from 1989 to 2011 (regression coefficient 0.3 g per year, 95%CI −2 g to +2.6 g).

**Table I:** Sample Sizes and LMS Parameters for Birth Weight in Down Syndrome by Sex and Gestational Age (n = 8,825)

Gestational age	Number of births		M			
(weeks)	Boys	Girls	Boys	Girls	L	S
28	11	10	960	937	−0.12	0.266
29	16	19	1132	1105	−0.03	0.253
30	26	23	1319	1288	0.06	0.243
31	29	27	1518	1482	0.15	0.237
32	73	41	1719	1678	0.23	0.233
33	84	54	1915	1869	0.31	0.228
34	117	85	2113	2063	0.39	0.220
35	192	156	2333	2277	0.47	0.206
36	414	298	2567	2506	0.55	0.189
37	693	525	2800	2733	0.62	0.174
38	1219	921	3019	2947	0.69	0.162
39	822	824	3164	3088	0.76	0.153
40	797	809	3251	3174	0.83	0.150
41	204	207	3304	3225	0.89	0.151
42	55	56	3318	3239	0.96	0.152
43	8	10	3300	3221	1.02	0.153

M, Median: L, skewness; S, coefficient of variation.

Tables II and III present the fitted birth weight centiles by sex and gestational age. At 38 weeks gestation, median weight was 2,567 g for boys and 2,506 g for girls.

**Table II:** Birth Weight (g) Centiles for Boys With Down Syndrome According to Gestational Age

Gestational age (weeks)	2nd	9th	25th	50th	75th	91st	98th
28	574	679	806	960	1149	1380	1665
29	685	809	957	1132	1341	1590	1887
30	805	951	1121	1319	1550	1819	2131
31	929	1098	1294	1518	1775	2068	2402
32	1049	1245	1467	1719	2003	2322	2678
33	1170	1390	1638	1915	2222	2561	2934
34	1304	1548	1817	2113	2437	2789	3170
35	1476	1739	2024	2333	2665	3020	3399
36	1682	1958	2253	2567	2899	3250	3618
37	1893	2181	2483	2800	3131	3476	3835
38	2092	2389	2698	3019	3350	3692	4044
39	2232	2534	2845	3164	3491	3826	4168
40	2302	2612	2929	3251	3580	3913	4252
41	2325	2647	2974	3304	3638	3976	4316
42	2317	2649	2983	3318	3655	3993	4332
43	2289	2627	2964	3300	3636	3971	4305

**Table III:** Birth Weight (g) Centiles for Girls With Down Syndrome According to Gestational Age

Gestational age (weeks)	2nd	9th	25th	50th	75th	91st	98th
28	560	662	786	937	1121	1347	1625
29	668	790	934	1105	1309	1552	1842
30	786	928	1094	1288	1513	1776	2080
31	906	1072	1263	1482	1733	2019	2344
32	1024	1215	1432	1678	1955	2266	2614
33	1142	1357	1599	1869	2169	2500	2864
34	1273	1511	1774	2063	2378	2722	3094
35	1441	1697	1976	2277	2601	2948	3318
36	1641	1911	2199	2506	2830	3172	3532
37	1848	2128	2424	2733	3056	3393	3744
38	2042	2332	2634	2947	3270	3604	3948
39	2179	2473	2777	3088	3408	3734	4068
40	2247	2550	2859	3174	3494	3820	4150
41	2270	2584	2903	3225	3552	3881	4213
42	2262	2586	2912	3239	3568	3898	4229
43	2234	2564	2893	3221	3549	3876	4202

**Figure 3:**
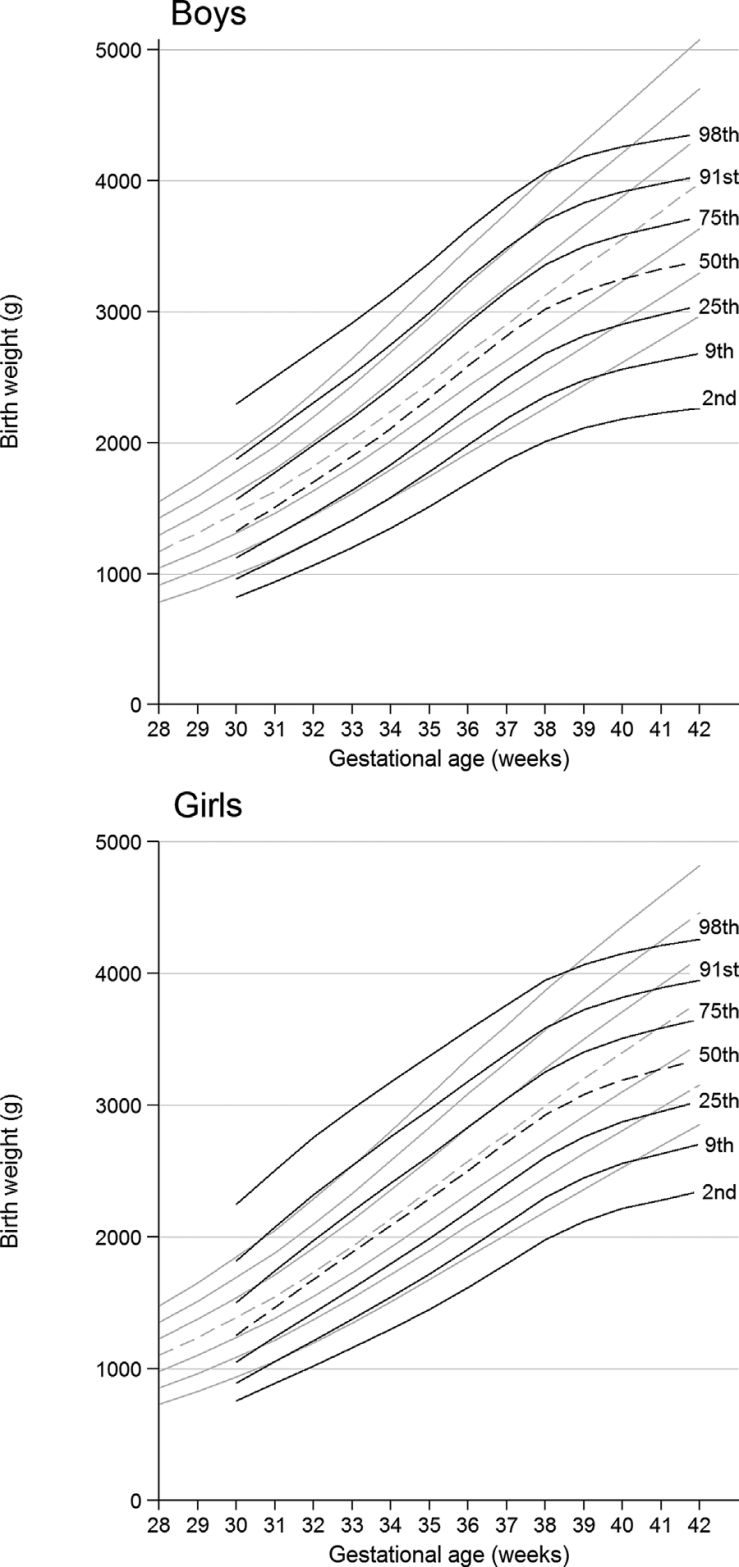
Birth weight centiles for boys and girls: Down syndrome (black lines) compared with revised UK‐WHO growth charts (gray lines) [Cole et al., [Link]].

Figure 3 shows the birth weight centiles by gestational age for babies with Down syndrome compared to unaffected babies. From 30 to 38 weeks median birth weight for Down syndrome babies was slightly but consistently lower than for unaffected babies. At 38 weeks the difference was 159 g for boys and 86 g for girls. But after 38 weeks the two median curves diverged, and by 40 weeks the shortfall was much greater (304 g for boys and 239 g for girls). The Down syndrome babies showed greater variation than the unaffected babies at all gestations, but particularly before 34 weeks when the centiles are positively skew, with a much wider gap between the 91st and 98th centiles than between the 2nd and 9th. This corresponds to the L value being well below one at early gestations.

**Figure 4:**
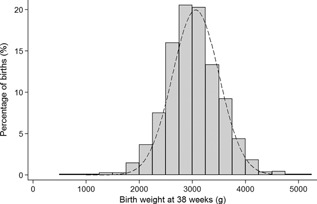
The distribution of birth weight at 38 weeks in babies with Down syndrome (grey bars) compared to unaffected babies (dashed line) [Office for National Statistics, 2012].

Figure 4 shows the distribution of birth weight at 38 weeks gestation for babies with Down syndrome compared to unaffected babies, with good agreement.

## DISCUSSION

Our study shows that babies with Down syndrome born near to term (39–41 weeks) were lighter than unaffected babies (Fig. 3). However the modal age at delivery was 38 weeks gestation (Fig. 2). This concurs with findings by Smith and McKeown [1955] and it was also shown though not mentioned by Clementi et al. [1990] and Boghossian et al. [2012]. Hence this is the first time in 60 years that attention has been drawn to the fact that modal gestational age in Down syndrome is 38 weeks, when mean birth weight was within 150 g of that for unaffected babies (Fig. 2). Hence there was little evidence of significant growth restriction in the first 38 weeks of pregnancy. After 38 weeks babies with Down syndrome were increasingly lighter than unaffected babies on average, suggesting that they were postmature and that intrauterine growth was slowing.

There is a relatively high rate of spontaneous fetal loss in Down syndrome pregnancies [Savva et al., 2006] with 3.3% of births being stillborn. The gestational‐age‐adjusted weight of stillborn babies was less than that of live births (data not shown), indicating that birth weight of live births is a biased measure of intrauterine growth, with the smaller fetuses being excluded. However, as the proportion of stillbirths did not alter from 38 to 42 weeks gestation, ignoring them would not explain the observed divergence in birth weight. So there is probably some intrauterine growth restriction amongst fetuses with Down syndrome at all gestations, but compared to unaffected fetuses it is greater from 38 weeks gestation.

The birth weight charts for US babies with Down syndrome derived by Boghossian et al. [2012] gave similar results to ours (e.g., at 38 weeks gestation median birth weight for boys was 3,048 g vs. 2,947 g in this study and for girls 3,092 g vs. 3,019 g). Median birth weight for unaffected babies is greater in North America than England and Wales, so a larger difference might have been expected. Against that, Boghossian only included babies admitted to hospital at birth or dying before admission; this might be expected to be a “sicker” population, biasing the weight centiles downwards, which might explain why the differences between the studies were small.

Heart anomalies are also relevant, as 44% of babies with Down syndrome have a heart anomaly [Morris et al., 2014]. Babies with heart anomalies are 100–200g lighter than unaffected babies born at the same gestational age [Rosenthal et al., 1991; Rosenthal, 1996]. This will account for some but not all of the difference in birth weight, and the observation of growth restriction after 38 weeks gestation remains relevant.

Smith and McKeown [1955] questioned whether the low birth weight of those with Down syndrome was due to shorter gestation or slower prenatal growth. In a study hampered by small numbers (n = 103) their difference in birth weight at 38 weeks was greater than in our study − 0.6 Lb (272 g). On this basis they concluded that there must be some intrauterine growth restriction prior to 38 weeks. They did however record placental weights and found these to be similar to those in their control population. Hence they suggested that the apparent growth restriction was likely to be due to a “lowered growth capacity of the foetus rather than inability of the intrauterine environment to support its growth”. However studies of first trimester growth restriction and aneuploidy using crown rump measurements [Bahado‐Singh et al., 1997; Schemmer et al., 1997] have shown that those with Down syndrome grow normally in the first trimester. Our own data suggest that from 30‐38 weeks the average intrauterine growth of those with Down syndrome differs little from that of other babies. Hence intrauterine growth restriction appears to be confined to gestations beyond 38 weeks.

In unaffected babies, slowing of intrauterine growth after the modal gestational age of 40 weeks is considered a surrogate marker for incipient postmaturity and signals a need for enhanced obstetric vigilance and possible intervention. For babies with Down syndrome, slowing of intrauterine growth appears to occur from the modal gestational age of 38 weeks, hence there may be an earlier onset of incipient postmaturity in this population and enhanced vigilance may be necessary from this time.

### Strengths and Weaknesses

Direct measures of intrauterine growth in late pregnancy are not available for babies with Down syndrome. Hence in our study and those of others [Clementi et al., 1990; Smith and McKeown, 1995; Boghossian et al., 2012] cross sectional birth weight data is used as a proxy measure. A strength of the study is the large sample size derived from a national register over 22 years with an estimated ascertainment rate of 93%. There was no evidence of a trend in birth weight over this time. A weakness is that 36% of the 13,940 live births recorded in the register had missing data for gestational age and/or birth weight. The register receives information from cytogenetic laboratories and then contacts the referral clinicians for further information. For some cytogenetic laboratories it is not possible to contact the referral clinicians and therefore the missing information is unlikely to be a source of bias as it is missing for administrative reasons. There was no association between gestational age and missing birth weight (*P* = 0.2). Mode of delivery was also unavailable and it may be that growth restriction was an indication for induced delivery before 38 weeks. However this is unlikely because mean birth weight was similar to that for unaffected babies up to 38 weeks. Detailed information on other associated anomalies (particularly heart anomalies) was not available.

### Implications for Perinatal Clinical Practice

#### Timing of elective delivery

Marlow has recently challenged the accepted view that for the general population the optimum time for delivery is 37–41 weeks gestation (full term) [Marlow, 2012]. He provides evidence of increased morbidity and mortality among those born in early term (37–38 weeks), agreeing with Clark that for the general population perinatal risk is a continuum for adverse outcomes that is minimal at 39–41 weeks of gestation [Clark and Fleischman, 2011]. Our findings suggest that in Down syndrome the optimum time for delivery may be earlier than for other babies, though there is currently no other evidence to support this. We suggest nevertheless that clinicians should be mindful of this possibility when a foetus with Down syndrome is still in utero at 40–41 weeks. In this situation they may wish to consider induction of labour. However, they need to balance this against the early weight gain/loss in newborns with DS, for which there is very little robust information.

There is a need for information about the associations between gestational age at delivery and short and long‐term outcome measures in babies with Down syndrome. Some of this might be available by linking existing cohorts and registers.

### Preterm Birth Weight Charts for Babies With Down Syndrome

The widely used UK‐WHO growth charts include a birth weight chart for 32–42 weeks gestation [Cole et al., [Link]]. Our findings show that median birth weight for those with Down syndrome is only slightly less than for UK‐WHO until 38 weeks gestation. However the centile lines are further apart, so there is a greater chance of Down syndrome babies being small or large for dates. We suggest that birth weights of preterm babies with Down syndrome should be plotted on the UK‐WHO charts up to 38 weeks gestation, and for later gestations at age 0 on the 2011 edition of the UK Down syndrome growth charts [Styles et al., 2002] (http://www.dsmig.org.uk/publications/growthchart.html). The distribution of birth weight in this study was similar to that of Styles et al (medians 3.06 and 3.00 kg respectively).

## CONCLUSION

The modal age at delivery in babies with Down syndrome is 38 weeks. For gestations up to 38 weeks their median birth weight is similar to that for unaffected babies, but after 38 weeks their median birth weight rises less fast than for unaffected babies. This may have implications for perinatal clinical practice.

## FUNDING

TJC was funded by UK Medical Research Council grant MR/M012069/1.
